# Individualized treatment with denosumab in children with osteogenesis imperfecta – follow up of a trial cohort

**DOI:** 10.1186/s13023-019-1197-z

**Published:** 2019-09-18

**Authors:** Heike Hoyer-Kuhn, Mirko Rehberg, Christian Netzer, Eckhard Schoenau, Oliver Semler

**Affiliations:** 1 Children’s Hospital, University Hospital Cologne, University of Cologne, Kerpener Straße 62, 50937 Cologne, Germany; 2 Institute of Human Genetics, University of Cologne, Cologne, Germany; 3 Cologne Centre for rare skeletal dysplasia in childhood, University of Cologne, Cologne, Germany

**Keywords:** Denosumab, Osteogenesis imperfecta, Bone mineral density, Mobility, Hypercalciuria

## Abstract

**Background:**

Osteogenesis imperfecta (OI) is a rare disease leading to hereditary bone fragility. Nearly 90% of cases are caused by mutations in the collagen genes *COL1A1/A2* (classical OI) leading to multiple fractures, scoliosis, short stature and nonskeletal findings as blue sclera, hypermobility of joints, bone pain and delayed motor function development. Bisphosphonates are used in most moderate and severely affected patients assuming that an increase of bone mineral density might reduce fractures and bone pain in patients with OI. Denosumab as a RANK ligand antibody inhibiting osteoclast maturation has been approved for osteoporosis treatment in adults. First data from small clinical trials promised a high efficacy of Denosumab in children with OI. Aim of this analysis was a retrospective evaluation of an individualized biomarker-associated treatment regime with Denosumab in 10 children with classical OI which were followed for 1 year after their participation in a pilot trial with Denosumab. Therefore urinary deoxypyridinoline levels were evaluated frequently as an osteoclastic activity marker and depending on that levels Denosumab injections were scheduled individually.

**Methods:**

Ten patients (age range: 6.16–12.13 years; all participated in the former OI-AK phase 2 trial (NCT01799798)) were included in the follow-up period. Denosumab was administered subcutaneously depending on the individual urinary excretion course of deoxypyridinoline (DPD/Crea) as osteoclastic activity marker with 1 mg/kg body weight. DPD/Crea levels were evaluated before denosumab administration and afterwards. If patients present after an initial decrease after injection with a re-increase up to the DPD/crea level before Denosumab injection next dosage was planned. Changes of areal bone mineral density (aBMD) using dual energy x-ray absorptiometry of the lumbar spine after 12 month was evaluated. Safety was assessed by bone metabolism markers and side effect reporting.

**Results:**

During follow-up mean relative change of lumbar aBMD was − 6.4%. Lumbar spine aBMD z-Scores decreased from − 1.01 ± 2.61 (mean ± SD) to − 1.91 ± 2.12 (*p* = 0.015). Mobility changed not significantly (GMFM-88 -6.49 ± 8.85% (*p* = 0.08). No severe side effects occurred. Dose intervals could be extended in the mean from 12 weeks previously to 20.3 weeks.

**Conclusions:**

On average, it was possible to prolong the intervals between drug administrations and to reduce the total dose about by 25% without a decrease of mobility or change of vertebral shape despite a reduction of lumbar aBMD during 1 year of biomarker-directed Denosumab treatment. Further trials are necessary to balance side effects and highest efficacy in children.

## Background

Osteogenesis imperfecta (OI) is a rare hereditary disease with an estimated incidence of 1:20,000. Main symptoms are fractures without adequate traumata, skeletal deformities, and scoliosis [1]. More than 85% of patients are affected by mutations in *COL1A1* or *COL1A2* impairing quantity and quality of collagen. Rare subtypes have been identified causing decreased bone mass due to alterations of posttranslational modification of collagen and changes in the extracellular matrix [2].

Despite different pathophysiologies most of the affected patients have been treated with antiresorptive drugs (e.g. bisphosphonates) to reduce osteoclastic activity [3]. Such a treatment has shown to increase bone mass. Different studies and the last version of the Cochrane review about the effects of bisphosphonates in OI showed ambiguous results regarding fracture rates [4, 5]. Because bisphosphonates are not approved for the use in children with OI, one major concern are possible long term side effects. Once given, bisphosphonates bind to the bone for years and might cause an adynamic skeleton in the end [6]. In 2010, Denosumab as a human IgG2 antibody that binds to RANK ligand was approved to treat osteoporosis in postmenopausal women [7]. By inhibiting the interaction of RANK ligand to its receptor RANK, Denosumab is a potent anti-resorptive agent, decreasing the differentiation of pre-osteoclasts and therefore reducing bone resorption and increasing bone mass [8]. Phase-3 trial in postmenopausal women comparing Denosumab and Alendronate showed a more powerful reduction of bone turnover markers and a higher increase of bone mineral density on denosumab compared to Alendronate [9]. Therefore it could be assumed that the beneficial effect is even higher comparable to a therapy with bisphosphonates in postmenopausal women [9]. Additionally, the subcutaneous application is more convenient and the potential risk of long term side effects might be reduced due to the complete degradation of the antibody after a few months [9].

Denosumab is neither approved in OI nor in children. Controlled trials about treatment intervals are still lacking. Rare case reports about Denosumab application in children with various skeletal diseases revealed severe side effects in some cases, especially after discontinuing treatment [10–13].

A first prospective trial was performed previously (NCT01799798) with Denosumab in children with OI by our group detecting a high efficacy of Denosumab in suppression of ostoclastic activity and increasing bone mineral density and mobility [14]. In the meantime a few reports have been published showing short time side effects in the calcium metabolism (suspected as rebound phenomenon) in adults and children.

Therefore the objective of this retrospective analysis was to evaluate the clinical course 12 months after end of the pilot trial of ten children with classical OI in an “individual biomarker-directed” treatment setting with Denosumab.

## Results

Ten children with a genetically confirmed OI (7 children with *COL1A1* and 3 children with *COL1A2* mutation) were included in the follow-up analyses. All patients have been treated within the former pilot trial for 48 weeks with Denosumab before entering the follow-up period. The analysed cohort included 7 males and 3 females with a mean age (±SD) of 8.60 years (±1.83). A synopsis of patient characteristics at start of the follow up period is given in Table 1.

**Table 1:** Baseline characteristics of the study cohort at the beginning of the follow up period

Participants [*n*]	10
Male [*n*] (%)	7 (70)
Age Mean [years] (range)	8.6 (6.16 – 12.13)
Height Mean [cm] (range)	110.1 (65.0 – 140.0)
Height Z-Scores ± SD	-4.53 ± 4.36
Weight Mean [kg] (range)	24.8 (7.8 – 30.1)
BMI Mean [kg/m^2^] (SEM)	18.33 (13.1 – 34.4)
OI Type 1/4 [*n*] (%)	8 (80)
OI Type 1/4 able to walk (GMFM item 69) *n* (%)	7 (70)
OI Type 3 [*n*] (%)	2 (20)
OI Type 3 able to walk (GMFM item 69) *n* (%)	0 (0)
Causative gene	
*COL1A1* [*n*] (%)	7 (70)
*COL1A2* [*n*] (%)	3 (30)

All patients have been examined in a clinically routine yearly checkup pattern approximately 1 year after end of the trial (53.04 weeks (± 6.30)). Eight out of ten patients received further Denosumab administration based on their individual urinary DPD/crea levels. Mean treatment interval (± SD) was 20.33 weeks (±4.17). One patient received after a minimum of 14 weeks the next dosage. Mean Height (± SD) increased from 110.1 cm (± 22.73) to 115.5 cm (± 24.19); *p* = 0.0001; (Z-scores − 4.53 ± 4.36 vs. -4.34 ± 4.61; *p* = 0.332).

### Bone mineral density

**Fig. 1:**
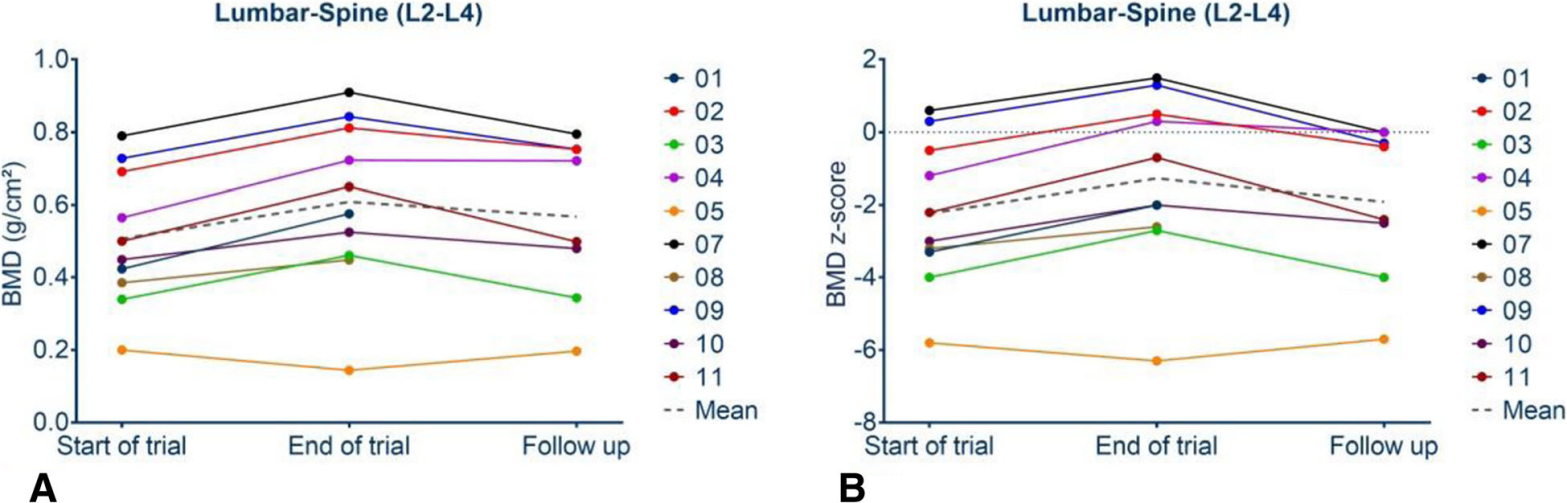
**a** Presents the individual absolute lumbar spine areal bone mineral density values (L2-L4) plotted against the period start of trial, end of trial, end of follow up period. In **b** age-adjusted z-scores and their change are shown individually and as mean for 8 patients between start of trial, end of trial, end of follow up period

Eight out of ten patients have been examined by DXA 12 months (53 ± 6.30 weeks) after end of the trial. Only these eight were included in the analysis. Absolute aBMD of the lumbar spine (L2 – L4) changed from 0.634 ± 0.251 g/cm^2^ to 0.568 ± 0.222 g/cm^2^ (mean ± SD; *p* = < 0.028) within the 1 year follow-up period (Fig. 1a, Table 2). Figure 1a presents absolute individual aBMD data over the trial and follow-up period. Z-scores decreased from − 1.01 ± 2.61 (mean ± SD) to − 1.91 ± 2.12 (*p* = 0.015) (Fig. 1b, Table 2). Individual and mean age- adjusted z-scores for lumbar aBMD of all patients are shown in Fig. 1b.

**Table 2:** Changes of areal bone mineral density, mobility, and height between start of trial, end of the trial and end of follow up period

	Number of patients	Start of trial	End of trial	End of follow up	*p*-value Start and end of trial	*p*-value End of trial and end of follow up	*p*-value End of follow up – End of trial Vs End of trial- Start of trial
aBMD lumbar vertebrae L2-L4 (g/cm^2^) (mean ± SD)	8	0.533 ± 0.202	0.634 ± 0.251	0.5676 ± 0.221	0.0041	0.028	0.0067
aBMD lumbar vertebrae L2-L4 z-score (mean ± SD)	8	−1.975 ± 2.217	− 1.013 ± 2.606	−1.913 ± 2.121	0.0037	0.015	0.0051
aBMD total body without head (g/cm^2^) (mean ± SD)	8	0.514 ± 0.116	0.587 ± 0.138	0.561 ± 0.129	0.0002	0.123	0.0035
aBMD total body without head z-score (mean ± SD)	8	−1.925 ± 1.624	−1.313 ± 1.755	−2.088 ± 1.54	0.0046	0.005	0.0036
Spine score (points) (mean ± SD)	9	24.33 ± 32.8	23.78 ± 34.64	20.33 ± 28.47	0.766	0.532	0.6415
GMFM 88 (%) (mean ± SD)	9	76.33 ± 33.58	78.83 ± 32.86	72.34 ± 34.75	0.198	0.077	0.0522
Height [cm] (mean ± SD)	9	106.4 ± 21	110.1 ± 22.7	115.5 ± 24.2	0.0004	0.0001	0.024
Height z-Scores (mean ± SD)	9	−4.467 ± 4.22	−4.533 ± 4.36	− 4.344 ± 4.61	0.6606	0.332	0.027

Absolute aBMD of the total body less head changed in the mean from 0.587 ± 0.138 g/cm^2^ to 0.561 ± 0.123 g/cm2 (*p* = 0.12) (mean ± SD) and age adjusted z-scores from − 1.31 ± 1.755 to − 2.10 ± 1.540 (*p* = 0.005) in the follow-up period (Table 2).

### Spine morphometry

Morphometry was assessed in 9 out of 10 patients. Using the “Koerber-score” the mean change of morphometry score was + 3.45 points, (*p* = 0.531) during the follow up period compared to an improvement of only 0.55 points in the first trial (*p* = 0.64) (Table 2).

### Mobility

**Fig. 2:**
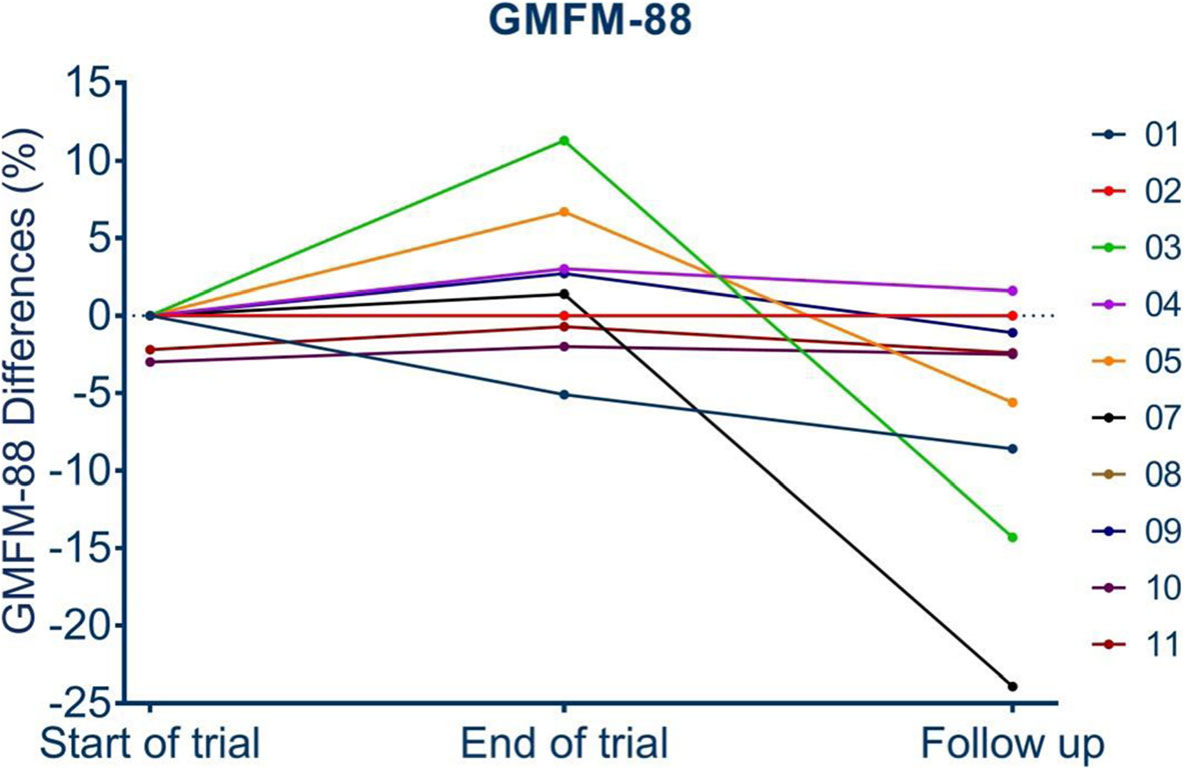
Demonstrates the individual urinary deoxypyridinoline excretion levels within the trial period and after end of the trial. Each Denosumab application is marked by an asterisk

Mobility did not change significantly in the trial cohort. Two patients were not assessed at the end of the follow-up period. One patient was treated by telescopic rod surgery 6 weeks before assessment and therefore was not able to perform the GMFM-88. The second patient was not available for a 12 months follow up assessment based on a trip to another country. Mobility results are presented in Table 2. Percentual changes of the individual mobility levels are presented in Fig. 2.

A mean change of motor function of − 6.49% (GMFM-88 score 78.83 ± 32.86% to 72.34 ± 34.75%; *p* = 0.077) was seen. Two patients finished the trial and the follow-up period with a full GMFM-88 score (100%). In these patients no change was detectable due to methodical issues.

### Changes of bone metabolism markers

**Fig. 3:**
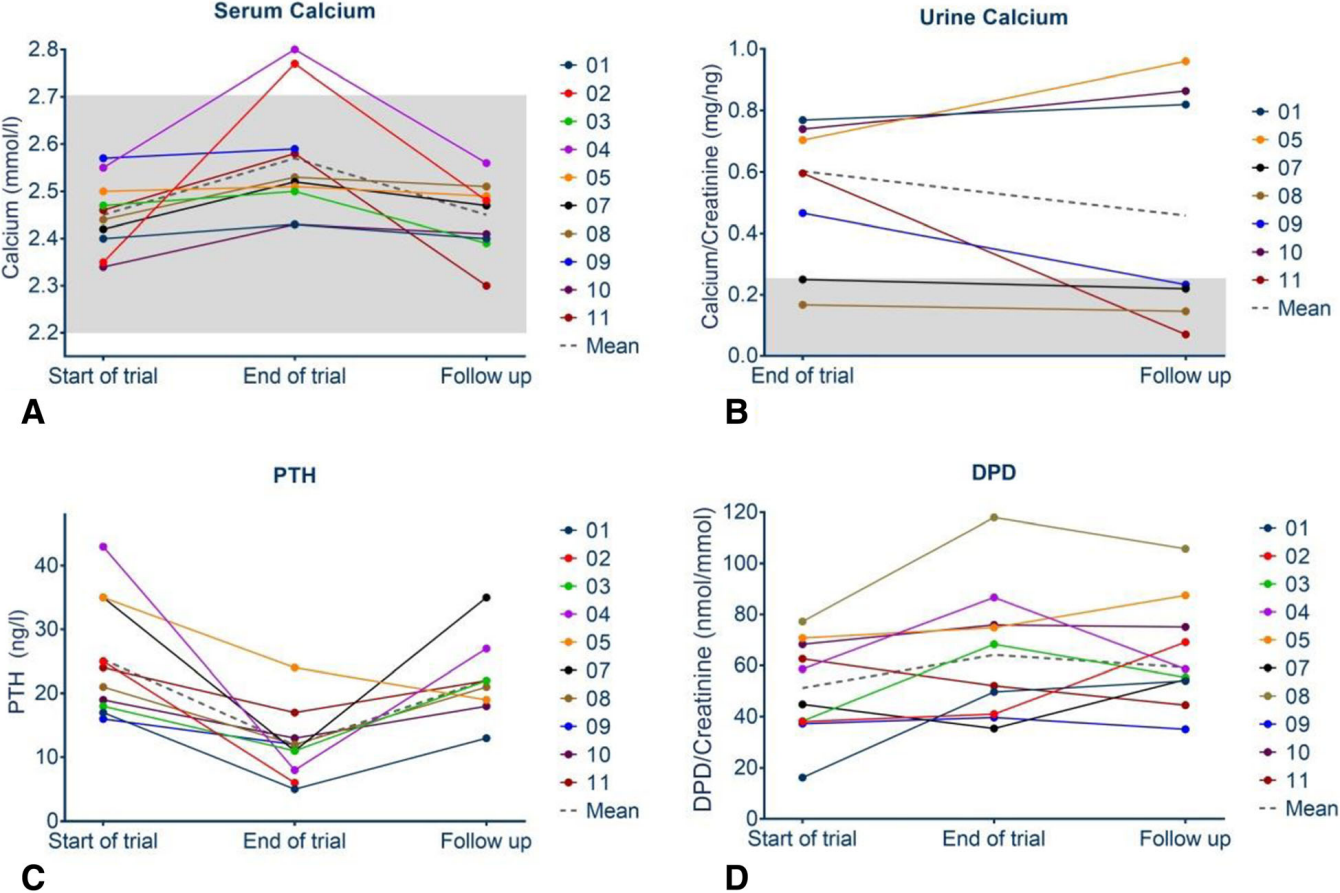
Shows the individual absolute differences of 10 study participants in the GMFM-88 assessments at start of trial, end of trial and end of follow up period. 2 children started with a maximum of 100 % in the GMFM-88 and maintained their mobility levels over the whole observation period. Therefore no changes are detectable in these 2 children (marked by the asterisk) lying on the dotted line which marks the line of no difference

**Fig. 4:**
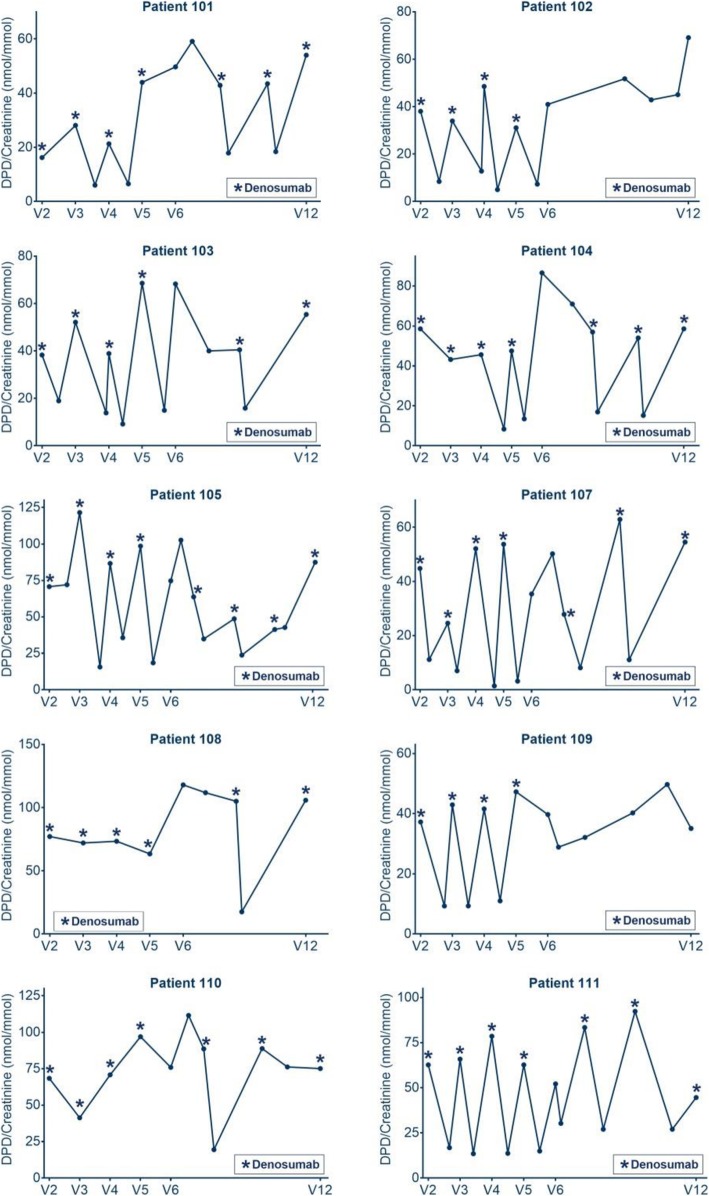
**a** Presents the assessed individual and mean serum calcium levels of ten patients over the observation period. **b** Presents the urinary calcium/creatinine excretion of seven patients out of spot urine samples after end of the trial. Presented are all data available from the whole observation period. In **c**, **d** individual and mean levels of 8 patients of the bone metabolism markers serum parathyroid hormone (PTH), and urinary deoxypyridinoline/creatinine (DPD/Crea) excretion at the different visits (start of trial, end of trial, follow up period) are demonstrated

Laboratory data are presented in Figs. 3a-d, 4, and Table 3. Mean urinary DPD levels had been constant within the follow up period from 58.17 ± 18.6 to 59.31 ± 15.84 nmol/mmol (mean ± SD) (*p* = 0.85). Serum calcium levels decreased in the mean from 2.56 ± 0.1344 to 2.44 ± 0.0779 (mean ± SD) mmol/l in the follow up period (*p* = 0.0039). PTH levels were suppressed at the end of the trial and increased from 12.63 ± 5.78 ng/l into the lower normal range of 22.13 ± 6.56 ng/l (mean ± SD) during the follow up (*p* = 0.0195).

**Table 3:** Mean changes of laboratory data between start of trial, end of trial and follow up period

Parameter	Number of patients	Start of trial	End of trial	End of follow up	*p*-value Start and end of trial	*p*-value End of trial and end of follow up
DPD/Krea (mmol/mmol) (mean ± SD)	9	48.33 ± 17.97	58.17 ± 18.6	59.31 ± 15.84	0.1149	0.8462
Serum Ca (mmol/l) (mean ± SD)	9	2.437 ± 0.068	2.563 ± 0.134	2.446 ± 0.078	0.0198	0.0174
PTH (ng/l) (mean ± SD)	8	26.50 ± 9.8	12.63 ± 5.8	22.13 ± 6.6	0.0066	0.0195

Vitamin D levels were analysed in 10 out of 10 children at V6 and in 9 out of 10 children at follow up: In 2 out of 19 analyses a vitamin D insufficiency with a level between 10 and 20 μg/l (25–50 nmol/l) (minimum 13.7 μg/l) was observed, no analysis revealed a level < 10 μg/l (25 nmol/l).

Urinary calcium/creatinine assessment was added to the evaluation after end of the trial based on upcoming concerns about rebound phenomena. In 7 children urinary calcium excretion was assessed, revealing an increased calcium excretion in spot urine samples at the end of the trial and after the follow up period in 8 out of 8 assessed patients who had continued the treatment with Denosumab.

### Safety

Application of Denosumab was well tolerated in general. Local pain during subcutaneous injection was reported by all patients. No discontinuation of medication was decided based on the detected and reported side effects within the 12 months after end of trial. One patient reported mild arthralgia 8–10 weeks after application of the drug. Pain resolved within 2 days. The child did request analgetic medication on 1 day with ibuprofen as a single dose. Two children reported general muscle pain/weakness 8–10 weeks after application.

Laboratory safety assessment revealed a mean calcium/creatinine excretion of 0.5274 ± 0.2415 mg/mg at end of the trial and 0.4733 mg/mg ± 0.3876 mg/mg at the end of the follow up period (*p* = 0.597). Serum calcium levels decreased in the mean from 2.56 ± 0.13 to 2.44 ± 0.07 mmol/l in the follow up period (*p* = 0.0174).

One severely affected patient presented at the 12 months follow up visit with a symptomatic hypercalciuria. He reported an episode of lower back pain 4 weeks before the visit without any referable cause. The renal ultrasound revealed urolithiasis with two concrements in the left kidney and sediment in the lower urinary tract. The stones consisted of calcium and the spot urine samples on 6 consecutive days revealed a hypercalciuria with a maximum of 3.8 mg calcium/ mg crea (reference range < 0.21). Renal function was not deteriorated (creatinine: 0.19 mg/dl) and remained within the reference range. Patient was advised to increase daily fluid intake and to start a stone metaphylaxis with alkaline citrate as concomitant medication. No further urolithiasis was detected in the follow up period.

## Discussion

Our analysis provides data about an individualized treatment approach with the osteoclast antibody Denosumab in children with OI. After a treatment period of 1 year with a fixed dose interval of 12 weeks the following doses were given based on changes of urinary bone resorption markers. Denosumab was administered when bone resorption markers increased. This approach allowed us to reduce the dose by 25% (24 injections in 8 patients instead of 32 during 12 months). In 2 additional patients we were able to stop treatment because bone resorption markers have not increased above reference ranges probably due to end of growth and reduction of bone metabolism in adolescents.

Denosumab is used in different indications in childhood without being approved in this age group at all. Dose and interval of treatment differ significantly [15]. Children with neoplastic disorders like giant cell tumors or giant cell granulomas were treated with 120 mg Denosumab monthly [16, 17]. Children with osteoporosis due to impaired muscle function with cerebral palsy were treated with low doses of 10 mg of Denosumab. A boy with spinal muscular atrophy was treated with a dose of 60 mg [18, 19]. In patients with a localized high turnover osteoporosis and destruction of the skeleton by cystic lesions Denosumab was also used to decrease bone turnover. In children with fibrous dysplasia, aneurysmatic bone cysts and juvenile paget disease Denosumab had been administered in doses ranging from 0.5 mg/kg/day up to 70 mg in intervals from monthly to every 7 months [10, 11, 20].

OI is classified as a high turnover osteoporosis and therefore might require high doses and short intervals. Our patients had previously received bisphosphonates which decrease bone turnover. Therefore we decided to administer the antibody every 3 months in the first trial [14]. Our data of the follow-up period show that Denosumab suppressed bone turnover for a longer period and that we could decrease the number of injections by 25%. Regarding the different outcome parameters a careful interpretation of the small sample revealed the following aspects:

Despite the stable laboratory findings for bone resorption markers our patients presented with a significant reduction of areal bone mineral density measured by DXA during the follow up year. Bone mass was still increased at the end of follow-up compared to the levels at start of the trial. The change of aBMD in the trial period was significantly higher than the change in the follow up period.

Assessment of spine morphometry revealed no new vertebral compression fractures. Vertebral shape further improved during follow up. The differences within the trial period versus the follow up period showed no significant change. This is in contrast to many reports in adults with osteoporosis where vertebral compression fractures were described when treatment was discontinued or treatment intervals have been prolonged [21].

The results of the mobility tests indicate that the therapeutic effect could be maintained throughout the follow up period. Due to the unchanged level of mobility and the absence of new vertebral fractures it could be speculated that only bone mass was resorbed which was not functionally relevant. This bone mass was accumulated during the first year of treatment with short interval of injections due to the complete suppression of bone remodelling. The restart of physiological remodelling was reflected by the increase of PTH into the normal range after suppression during the trial year.

Growth was not influenced in our patients as demonstrated by a constant z-score throughout the 2-year observation period.

Many reports about calcium homoeostasis in patients treated with Denosumab have been published recently. The risk of hypocalcemia during the first 2–4 weeks after injection could be compensated in our patients by oral calcium substitution. Recently a rebound hypercalcemia after Denosumab effect ceased has become a reason of concern [22–24]. We monitored calcium excretion and detected a reduction of hypercalcemia in those patients which were sufficiently treated with longer intervals between Denosumab injections. In those patients the rebound seems to be less severe than in patients treated more frequently.

No longterm data about the risk of nephrocalcinosis or calcification of coronary arteries later in life in OI-patients with hypercalcemia or hypercalciuria are available. Even if the levels remained within the reference ranges in our patients there still might be an increased risk in patients with fluctuating serum and urinary calcium levels. Therefore calcium excretion and serum calcium levels at the end of the treatment intervals should be monitored. This is especially important in children but should also be considered in adults after discontinuation of Denosumab.

Altogether these data show the possibility of an individualized treatment approach based on urinary bone resorption markers. In seven out of eight patients total dose per year of Denosumab could be reduced without impairments of mobility or vertebral shape. Our data show that even with this reduced total dose and frequency the magnitude of mobility changes and vertebral morphology seems to be comparable between the trial period and the follow up period (no statistical significant differences).

Our study is limited by the small number of patients and by the heterogeneity of the phenotypes. None the less this is the first report about dosing intervals of Denosumab in children due to our knowledge.

## Conclusion

In summary, this report about an individualized treatment approach with Denosumab in children with OI gives evidence

1. that it was possible to individualize treatment intervals based on urinary bone resorption markers in children with OI.
2. that the interval between injections could be extended compared to the previous treatment without increasing vertebral fracture rate or reducing mobility, especially in patients with mild and moderate types of OI.
3. that areal bone mineral density decreased in patients with a prolonged interval without causing clinical impairments.
4. that bone resorption increased rapidly after degradation of Denosumab causing hypercalciuria.
5. that serum calcium homoeostasis needs to be carefully monitored in the future to better assess the risk of calcifications in children and adolescents treated with Denosumab.

## Materials and methods

Ten children with genetically confirmed OI which participated in the earlier performed pilot trial were included in this follow up study. After end of the OI-AK trial a change of therapeutic approach to the former bisphosphonate treatment was offered to all families and children (all patients had at least a 2 year course of neridronate before entering the denosumab trial – this was an inclusion criterium). In an individual discussion with the parents and the patient at the end of the trial about efficacy, side effects, and the individual pros and cons while receiving Denosumab risks and benefits have been outweighed and a decision about further treatment and follow up regimen was made. The retrospective analysis was performed over a 12 months follow up period after the individual end of the trial. At the end of trial it was reanalyzed if the following exclusion criteria for further Denosumab administrations were still absent: hypocalcemia (< 1.03 mmol/l ionisized Calcium); reduced renal function (estimated GFR (Schwartz formula) < 30 ml/min/1.73m^2^); current treatment with other osteoanabolic or antiresorptive drugs. In- and exclusion criteria for Denosumab treatment were published in 2016 [14]. Patient characteristics at start of the follow up period are described in Table 1.

Denosumab treatment was performed in an individualized concept meaning that the treatment schedule was individualized depending on the urinary DPD/crea excretion course. Recovery of osteoclastic activity was assessed by bi-weekly measurement of urinary deoxypyridinoline/creatinine ratio (DPD/crea) in spot urine. Increases to the DPD/crea level before the last Denosumab injection were defined as a recovery of osteoclastic activity and therefore end of bone resorption suppression by the agent.

According to the earlier trial protocol Denosumab dosage was chosen and concomitant medication was prescribed. Denosumab (Prolia®, Amgen Inc., Thousand Oaks, CA) was administered with 1 mg per kg body weight subcutaneously. Additionally, every patient received post injection (p.i.) weight adjusted oral calcium and vitamin D supplementation:

- < 15 kg body weight day 0–14 p.i.: 2 × 250 mg/ day Ca, day 15–28 p.i. 1 × 250 mg /day Ca, day 0–28 p.i. 500 IE Vit D
- 15–30 kg body weight day 0–14 p.i.: 2 × 500 mg/ day Ca, day 15–28 p.i. 1 × 500 mg /day Ca, day 0–28 p.i. 500 IE Vit D
- > 30 kg body weight day 0–14 p.i.: 2 × 1000 mg/ day Ca, day 15–28 p.i. 1 × 1000 mg /day Ca, day 0–28 p.i. 1000 IE Vit D

Primary objective was to follow the relative change of areal bone mineral density (aBMD) of the lumbar spine (L2-L4) after an individualized treatment of 12 months with Denosumab compared to end of the trial. aBMD was assessed using a GE Lunar iDXA densitometer (GE Ultraschall GmbH, Germany) and software version Lunar iDXA 14.10 for the lumbar spine (L2–L4) and for the total body less head (TBLH). aBMD results were transformed to age-specific z-scores using reference data provided by the manufacturer [25]. Quality checks are performed at least weekly based on the local authority requirements and revealed a precision variability of 0.23% between the phantom measurements. To reduce radiation dosage DXA scans were performed every 12 months in clinical routine. Secondary, whole body DXA measurements were performed at 12 months intervals to evaluate the total body less head aBMD.

Radiographs (Philips Optimus 65 Bucky Diagnostic TH and VT Philips Healthcare, The Netherlands) of the lumbar and thoracic spine were taken once a year (yearly checkup) in a lateral direction in a spine dedicated technique. Spine morphometry was evaluated based on the semi-quantitative score described by Koerber et al. 2011. This numeric score include compression of vertebrae of the thoracic and lumbar spine separately, as well as the shape of deformities (e.g. fish-shape or wedge shape) in these regions and the kyphosis of the whole spine. The score was developed to quantify impairments of the vertebrae in a semiquantitative way allowing the detection of smaller changes of morphometry compared to other more generalized scores like the Genant grading [26–28]. Especially for follow-up examinations, the underlying concept of the “Severity Classification” is extended to a much more detailed “Severity Score”. This uses a larger range of numbers (1–138) describing the overall severity more detailed, allowing a further refined assessment of the actual status and occurring changes during treatment.

Bone metabolism markers were assessed in the blood at least at each visit. Parathyroid hormone (Cobas C 702 (Roche Diagnostics), Germany, reference range 15–65 ng/l), 25-OH-Vitamin D (Cobas C 702 (Roche Diagnostics), Germany, reference range 30–70 μg/l) and total serum calcium (Cobas C 702 (Roche Diagnostics), Germany, reference range 2.2–2.7 mmol/l) were measured in the serum by our central laboratory. Urinary deoxypyridinoline/creatinine ratio (DPD/crea) of the second morning urine was used to monitor bone resorption measured with High –Performance –Liquid -Chromatography with age matched reference data. Urinary calcium levels (mmol/l) and urinary creatinine levels (mg/dl) were measured in spot urine (second morning sample, Cobas C 702 (Roche Diagnostics)), Germany,) by our central laboratory. Urinary calcium/creatinine (ca/crea [mg/mg]) excretion was calculated afterwards ((calcium (mmol/l) × 4): creatinine (mg/dl) = calcium/creatinine (mg/mg)); normal range ca/crea excretion in children < 0.21 mg/mg) [29].

Mobility was analyzed using the gross motor function measurement (GMFM-88) every 12 months [30].

Height and weight were measured at least every 12 months. Height/length were measured either using a stadiometer or lying on a bench for children not able to stand. All patients were measured with the same method during the assessment period. Body weight was measured using a sitting scale.

Treatment of the patients was conducted in accordance with the principles of the Declaration of Helsinki and was approved by the ethics committee (approval number: 12–283).

## Statistics

All analyses were performed using the full intention-to-treat set including all available patients. Individual and mean changes over time in the various outcome variables were displayed graphically. The mean change in lumbar bone mineral density at the end of follow up period and the mean change in aBMD z-score were calculated with a 95% confidence interval and tested for significance using the paired t-test. Analogous methods were employed for mobility, laboratory, and auxiologic variables as appropriate. Additionally, magnitude of changes between the trial period and the follow up period were compared by calculation with a 95% confidence interval and tested for significance using the paired t-test. Cumulative lists of adverse events (AEs) and serious adverse events (SAE’s) were presented descriptively. If a patient could not perform a mobility test based on a clinical contraindication, he was excluded a-priori from the analyses. No subgroup analysis of the gender groups was performed due to the small sample size. *P*-values < 0.05 were considered significant. Statistical analyses were conducted using GraphPad Prism 6.05.

## Data Availability

Data are stored and available at the center according to the national laws.

## Abbreviations

aBMD: Areal bone mineral density
DPD: Deoxypyridinoline
GMFM: Gross motor function measurement test
OI: Osteogenesis imperfecta
SD: Standard deviation
